# Proteome Regulation Patterns Determine Escherichia coli Wild-Type and Mutant Phenotypes

**DOI:** 10.1128/mSystems.00625-20

**Published:** 2021-03-09

**Authors:** Tobias B. Alter, Lars M. Blank, Birgitta E. Ebert

**Affiliations:** a Institute of Applied Microbiology (iAMB), Aachen Biology and Biotechnology (ABBt), RWTH Aachen University, Aachen, Germany; b Australian Institute for Bioengineering and Nanotechnology (AIBN), The University of Queensland, Brisbane, Australia; Chan Zuckerberg Biohub

**Keywords:** constraint-based modeling, enzyme kinetics, metabolic engineering, protein allocation, transcriptional control, *Escherichia coli*

## Abstract

It is generally recognized that proteins constitute the key cellular component in shaping microbial phenotypes. Due to limited cellular resources and space, optimal allocation of proteins is crucial for microbes to facilitate maximum proliferation rates while allowing a flexible response to environmental changes. To account for the growth condition-dependent proteome in the constraint-based metabolic modeling of Escherichia coli, we consolidated a coarse-grained protein allocation approach with the explicit consideration of enzymatic constraints on reaction fluxes. Besides representing physiologically relevant wild-type phenotypes and flux distributions, the resulting protein allocation model (PAM) advances the predictability of the metabolic responses to genetic perturbations. A main driver of mutant phenotypes was ascribed to inherited regulation patterns in protein distribution among metabolic enzymes. Moreover, the PAM correctly reflected metabolic responses to an augmented protein burden imposed by the heterologous expression of green fluorescent protein. In summary, we were able to model the effects of important and frequently applied metabolic engineering approaches on microbial metabolism. Therefore, we want to promote the integration of protein allocation constraints into classical constraint-based models to foster their predictive capabilities and application for strain analysis and engineering purposes.

**IMPORTANCE** Predictive metabolic models are important, e.g., for generating biological knowledge and designing microbes with superior performance for target compound production. Yet today’s whole-cell models either show insufficient predictive capabilities or are computationally too expensive to be applied to metabolic engineering purposes. By linking the inherent genotype-phenotype relationship to a complete representation of the proteome, the PAM advances the accuracy of simulated phenotypes and intracellular flux distributions of E. coli. Being equally computationally lightweight as classical stoichiometric models and allowing for the application of established *in silico* tools, the PAM and related simulation approaches will foster the use of a model-driven metabolic research. Applications range from the investigation of mechanisms of microbial evolution to the determination of optimal strain design strategies in metabolic engineering, thus supporting basic scientists and engineers alike.

## INTRODUCTION

For many decades, metabolic models have been developed to describe, unravel, and understand the drivers of microbial phenotypes. In their simplest forms, these models quantitatively connect observable phenomena such as carbon source consumption and biomass formation, leading to seminal empirical growth laws such as the Monod equation (1). In general, coarse-grained models aid in explaining the dependencies between intracellular processes and corresponding phenotypes (2–7). Constraint-based modeling techniques facilitate the prediction of growth rates and by-product secretion, as well as the investigation of metabolic flux distributions solely based on the stoichiometry of biochemical reaction networks and an appropriate cellular objective function (8–12). The development of genome-scale constraint-based models (GEM) fostered the investigation of fundamental biological phenomena (9, 13, 14), the systematic analysis of complex omics data sets (15–18) and the suggestion of favorable genetic perturbations for the overproduction of desired chemicals (19–23).

The utilization of GEMs has made valuable contributions to systems biological analyses and metabolic engineering. However, the GEM’s predictive capabilities of cellular phenotypes strongly rely on *ad hoc* capacity bounds on key reactions (15, 24), without which basic phenomena such as overflow metabolism are not observable *in silico*. The consideration of additional cellular processes and properties in metabolic reconstructions resolved these predictive insufficiencies of GEMs. In this manner, macromolecular expression (ME) models couple metabolism to gene expression by linking enzyme concentrations to metabolic reactions and accounting for the transcriptional and translational processes leading to enzyme expression (25). ME models simultaneously simulate maximum growth and substrate uptake rates and the underlying responses on the mRNA level, as well as the corresponding gene expression profiles at metabolic steady state (26). Thus, ME models facilitate holistic insights into intracellular processes and how they are affected by environmental, biochemical, or genetic perturbations (27–29), while reliably informing about corresponding flux distributions. As advantageous ME models are for correct predictions at the flux or phenotypic level, their detail and complexity can be cumbersome for future applications in strain design approaches. Many other approaches exist that add various resource or spatial constraints to classical stoichiometric models to investigate otherwise undisclosed growth laws (30–36). However, most of them are either similarly computationally expensive or demand a tremendous number of mostly unknown parameters.

Cellular protein allocation and its regulation have previously been suggested as the main drivers of metabolic phenomena and a key process behind bacterial growth laws (3, 5–7, 37–39). The incorporation of protein constraints in GEMs exploits the principles of protein allocation as a fundamental growth law. It simultaneously allows for the use of established, tractable, and intuitive constraint-based modeling methods. By dividing the limited proteome into three growth-variant sectors representing (i) ribosomal proteins, (ii) anabolic enzymes, and (iii) catabolic enzymes and one invariant housekeeping protein sector, the constrained allocation flux balance analysis (CAFBA) framework computes the optimal partitioning between these protein sectors and the correspondingly weighted flux rates to reach maximum growth (37, 39). Thus, CAFBA accounts for the trade-off between a limited protein availability for biosynthesis and growth and enables a quantitative prediction of pathway usages under various conditions, particularly suboptimal growth yields during overflow metabolism. The integration of enzyme kinetics into a GEM of Saccharomyces cerevisiae and Escherichia coli in the form of explicit enzymatic constraints on flux rates facilitated the utilization of proteomics data (40). The respective GECKO framework (GEM with enzymatic constraints using kinetic and omics data) gives detailed insights into metabolic realizations based on proteome measurements and predicts growth phenomena even without augmented protein concentration data.

Here, we introduce an approach that consolidates protein allocation and enzymatic constraints on metabolic fluxes of an E. coli GEM. The resulting protein allocation model (PAM) represents the major protein sectors and their various ratios with changing growth conditions (41). The PAM predicts experimentally observed phenotypes and intracellular flux distributions at maximum growth and on various growth media. Besides the wild-type behavior, the PAM’s predictive ability is demonstrated for strains harboring gene deletions or expressing heterologous proteins. In line with previous studies, we emphasize the fundamental role of protein allocation in steering microbial metabolism. Therefore, the PAM framework is envisioned as a practical approach to improve the rational design of microbial production strains, as it enables rapid dry-lab screening of design and cultivation strategies with improved reliability.

## RESULTS

### Accounting for the total proteome in genome-scale metabolic models.

A key challenge for microbes is distributing the limited proteins to the intracellular processes in a way that allows for maximum growth under a given environmental condition. While the concentration of ribosomes efficiently meets the demand of protein biosynthesis under mildly nutrient-limited to unlimited conditions (5, 42, 43), the protein household for energy and biomass precursor production generally contains unutilized and underutilized enzymes (44) to allow for a flexible response to environmental changes. Three major protein sectors cover this condition-dependent proteome and represent (i) translational protein, including ribosomes, (ii) metabolically active enzymes, and (iii) un- as well as underutilized enzymes (3). We will refer to the latter as the unused enzyme sector for simplicity. The fourth protein sector covers the housekeeping proteins, whose abundance is constant under any growth condition. Consequently, this sector cannot cause condition-dependent phenotypes. The biomass synthesis equation covers its constant demand, but the protein allocation toward the housekeeping sector does not need to be modeled separately.

**FIG 1:**
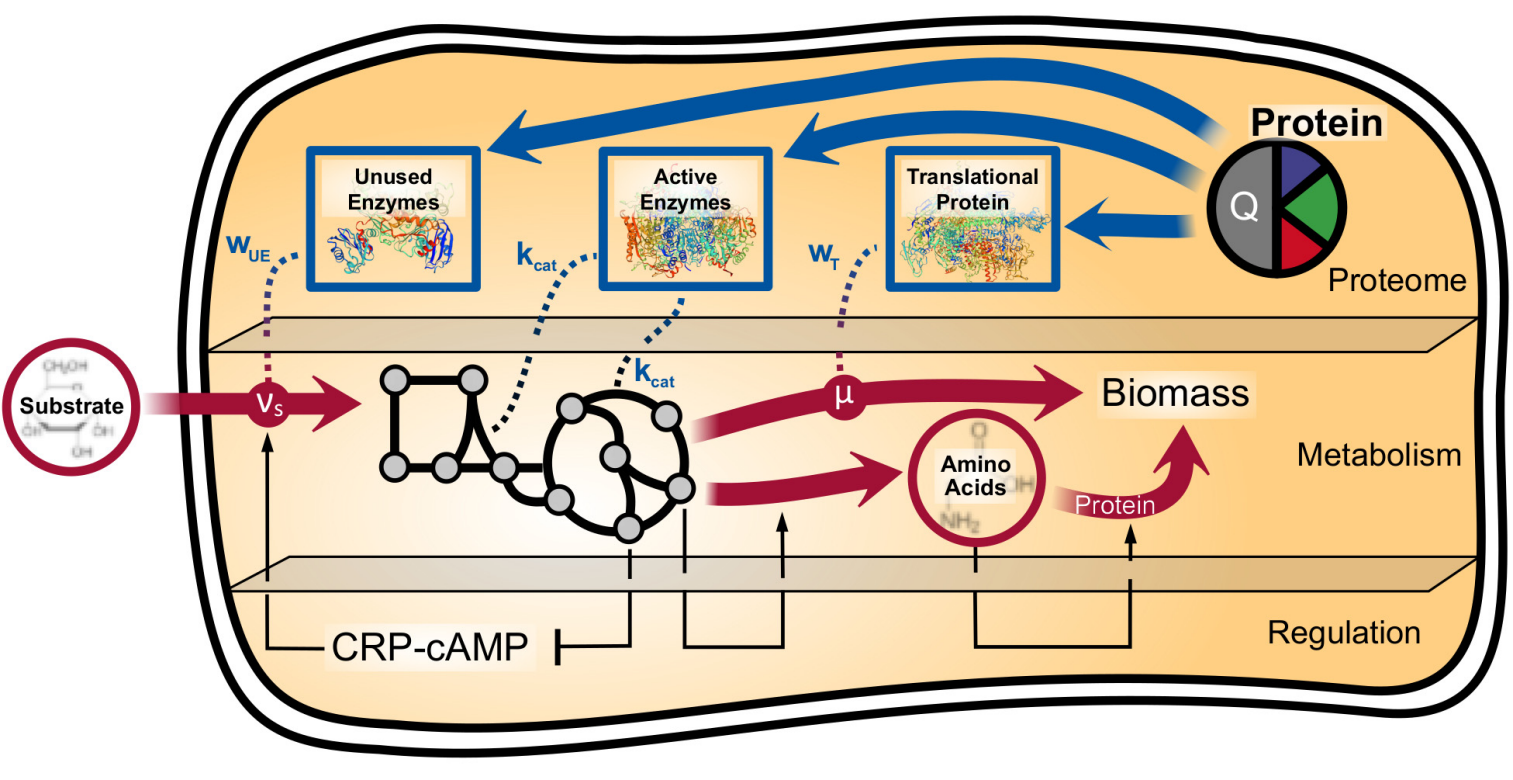
Scheme of the protein allocation model, including the classical metabolic as well as the added proteome level. *w*_T_, *w*_UE_, and *k*_cat_ represent the linear correlation factors between model variables and the translational, unused enzyme, and active enzyme sectors, respectively. Q denotes the housekeeping protein sector, whose concentration is constant. The dotted lines mark the model-inherent, linear relations between protein sectors and metabolic rates. A simplified mechanism regulating the microbial proteome, which is not part of the model, is also shown in accordance with the theory proposed in reference 4. It depicts the activation of catabolic enzyme expressions by the CRP-cAMP complex, whose synthesis is hampered by elevated amino acid precursor levels. Additionally, the activating effects of the concentration of amino acids and their precursors on protein and amino acid synthesis, respectively, are sketched.

To quantitatively account for the condition-dependent protein allocation, we modeled and added each relevant protein sector independently to the E. coli K-12 MG1655 GEM *i*ML1515 (45) (Fig. 1). We refer to the resulting model as the protein allocation model (PAM). The mass concentration, *ϕ*, of each sector is a linear function of one or more inherited variables or fluxes of *i*ML1515. They have been partly fitted to experimental proteomic data as depicted in Table 1 and are described in detail in Materials and Methods.

**TABLE 1:** Modeled mass concentrations *ϕ* of protein sectors and their linear dependencies on inherited variables of the E. coli K-12 MG1655 GEM *i*ML1515^*a*^

Protein mass concn	Symbol	Linear dependency
Active enzymes	*ϕ*_AE_	Flux rates *ν* of metabolic reactions
Unused enzymes	*ϕ*_UE_	Substrate uptake rate *ν*_s_
Translational protein	*ϕ*_T_	Growth rate *μ*
Total protein	*ϕ*_P,c_	*ϕ*_AE_, *ϕ*_UE_, *ϕ*_T_

a Parameters within these linear dependencies and their (data) sources are listed in Table 2.

### The active enzyme sector.

The active enzymes sector covers the protein demand of all enzymatic reactions of the *i*ML1515 model in a GECKO fashion (40). GECKO introduces enzyme mass balances to classical stoichiometric models and couples the manifestation of metabolic fluxes to enzyme abundances. That is, each modeled enzyme concentration within this sector is linearly dependent on the flux rate of the catalyzed metabolic reaction. The linear relations are based on a simplified rate law of reversible Michaelis-Menten reactions, thus on simple mass action kinetics, which neglects the nonlinear reversibility, enzyme saturation, and regulation factors (38). With this simplification, enzymes are assumed to operate at their maximal velocity, at which the turnover number *k*_cat_ describes the relation between enzyme concentration and flux rate. Hence, *in silico* metabolic fluxes are only limited by the enzyme’s maximum capacity. While this formulation can lead to biologically more feasible solutions (40), enzymes do not generally operate at their maximum velocity (46, 47). Therefore, in the PAM context, the turnover number describes an enzyme’s maximum capacity within a metabolic network operating under unlimited growth conditions, that is, under maximum growth and substrate uptake rate. The additional protein burden due to an incompletely used capacity of enzymes, e.g., under carbon-limited growth conditions, is accounted for by the unused enzyme sector introduced in the following section.

### The unused enzyme sector.

Particularly under nutrient-limited conditions, microbial cells put enzymes on hold to react quickly to changing environmental conditions (27, 44). This behavior contrasts with the general assumption that cells make efficient use of limited resources but reflects a necessary trade-off between maximal resource efficiency and quick adaptability. However, reversible enzyme kinetics prohibit the simultaneous manifestation of maximum velocity for all enzymes since the substrate of one enzyme is the product of another, inflicting opposing driving forces on both enzymes (38). As shown by metabolomics analyses, enzymes operate at substrate concentrations in their K_M_ range under most physiological situations, thus well below their maximum velocity, and are considered underutilized (46). Moreover, unutilized enzymes of inactive, mostly catabolic metabolic pathways may be expressed to allow for swift metabolic adjustments upon changes in the environmental conditions or substrate availability.

**FIG 2:**
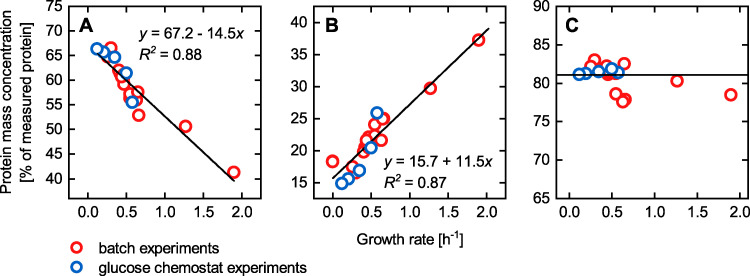
Protein mass data of distinct protein sectors under diverse growth conditions taken from Schmidt et al. (41). (A) All proteins represented by the *i*ML1515 GEM and found in the proteomic data set, comprising the active and unused enzymes sectors. (B) Translational sector covering proteins assigned to the COG (Clusters of Orthologous Groups) class “translation, ribosomal structure, and biogenesis,” which are not included in the *i*ML1515. The black lines in panels A and B are linear fits of the data points resulting in the shown equations and coefficients of determination *R*^2^. (C) Sum of the protein mass concentrations shown in panels A and B. The horizontal black line marks the 81% of measured protein mass used to constrain protein availability in the PAM. Glucose chemostat and batch experiments are highlighted in blue and red, respectively. Note that protein concentrations relative to the cell dry weight (cdw) were related to the total protein concentration of 0.32 g ${\text{g}}_{\text{cdw}}^{-1}$ measured by Schmidt et al. (41).

With a detailed analysis of experimental proteomic data of E. coli (41), O’Brien et al. (44) showed the existence of such under- and unutilized enzymes, which we summarized in the PAM as the unused enzyme sector. More specifically, O’Brien augmented an E. coli ME model with a comprehensive proteomic data set covering all enzymes of the *i*ML1515 model (Fig. 2A) and assessed the environment-specific proteome utilization *in silico*. They showed that the mass concentration of the active enzyme sector decreases with increasing growth rates, despite the increased metabolic activity and rising protein requirements. Conversely, enzymes, which are not catalytically active, accumulate the stronger the carbon limitation and the lower the growth rate. This phenomenon is regulated by the cAMP signaling pathway, which senses the carbon influx (4). We linked the resulting negative feedback loop between protein expression and substrate uptake caused by the cAMP signaling pathway (Fig. 1) to the functional representation of the unused enzyme sector (see Materials and Methods for details). As a result, protein allocation toward the unused enzyme sector decreases with increasing substrate uptake rate and diminishes at the maximum substrate uptake rate. In this way, the PAM allows for the adaption of substrate-specific allocation characteristics of the unused enzyme sector.

### The translational protein sector.

The quantitative description of the translational protein sector was empirically derived from global E. coli proteome measurements. The experimental proteomic data set was taken from Schmidt et al. (41), who investigated the quantitative dependencies between protein allocation and growth for a wide range of conditions, substrates, and different E. coli strains. We processed the measured protein mass data (fg cell^−1^) to arrive at model-relevant mass units relative to the cell dry weight (cdw) (g ${\text{g}}_{\text{cdw}}^{-1}$). We found that the translational protein mass concentration correlates linearly with the growth rate and modeled this protein sector accordingly (Fig. 2B). This linear growth dependency depicts a resource-efficient regulation of the translational apparatus to justly provide the proteins necessary for maintaining maximal division rates in a particular environmental condition, as reported previously (48, 49).

### The total protein concentration constraint.

Under most conditions, the total protein content of an E. coli cell is approximately 0.55 g ${\text{g}}_{\text{cdw}}^{-1}$ (50). A proteomic analysis of various E. coli strains grown under different conditions (41) covered a constant protein mass concentration of 0.32 g ${\text{g}}_{\text{cdw}}^{-1}$ (58% of the total protein content) across conditions. Therefore, a constant 81%, or 0.26 g ${\text{g}}_{\text{cdw}}^{-1}$ (*ϕ*_P,c_), is allocated to the three protein sectors, that is, the active enzymes, unused enzymes, and translational protein sectors (Fig. 2C). The unassigned proteins of the proteomic data set and those not covered by the experimental data were assigned to the housekeeping protein sector. The protein fraction *ϕ*_P,c_ of 0.26 g ${\text{g}}_{\text{cdw}}^{-1}$ considered by the PAM is in agreement with the fact that the growth-dependent part of the proteome constitutes roughly half of E. coli’s total protein content (3–5, 37, 39, 51). Note that the metabolic cost of the total protein biosynthesis in terms of energy and amino acid demand is fully covered by the biomass equation inherited from *i*ML1515.

The proteins within the data sets of Schmidt et al. (41) not covered by the PAM are mainly poorly characterized proteins (51% of all the protein fractions within the proteomic data set not covered by the PAM) or are assigned to transcription (8%), replication (6%), and posttranslational modification (6%). In turn, approximately one-third of the gene products considered by the PAM have not been quantified by Schmidt et al. (41). Those proteins are mainly inner and outer membrane proteins (58% of all gene products in the PAM not covered by the proteomic data set) or allocated to the glycerophospholipid (9%) and alternate carbon (8%) metabolism.

As experimental proteomic data suggest a consistent protein mass concentration across conditions, we modeled and added the invariable sum of active enzymes, unused enzymes, and translation protein to the PAM:

(1) $$\phi_{\text{P,c}}=\phi_{\text{T}}\;+\;\phi_{\text{AE}}\;+\;\phi_{\text{UE}}=0.26\;\text{g}\;{\text{g}}_{\text{cdw}}^{-1}$$

Here, *ϕ*_T_, *ϕ*_AE_, and *ϕ*_UE_ are the variable protein mass fractions of the translational protein, active enzymes, and unused enzyme sector, respectively. *ϕ*_P,c_ is the constant total protein mass concentration. Equation 1 introduces a concise representation of the capabilities and limits of E. coli’s metabolism and its condition-dependent protein allocation to stoichiometric modeling approaches. Due to the linear nature of the added protein allocation and enzyme activity constraints, classical flux balance analysis (FBA) and other established constraint-based modeling approaches can be applied to the PAM without a significant loss in computation speed compared to purely stoichiometric models.

### The protein allocation model predicts wild-type phenotypes.

**FIG 3:**
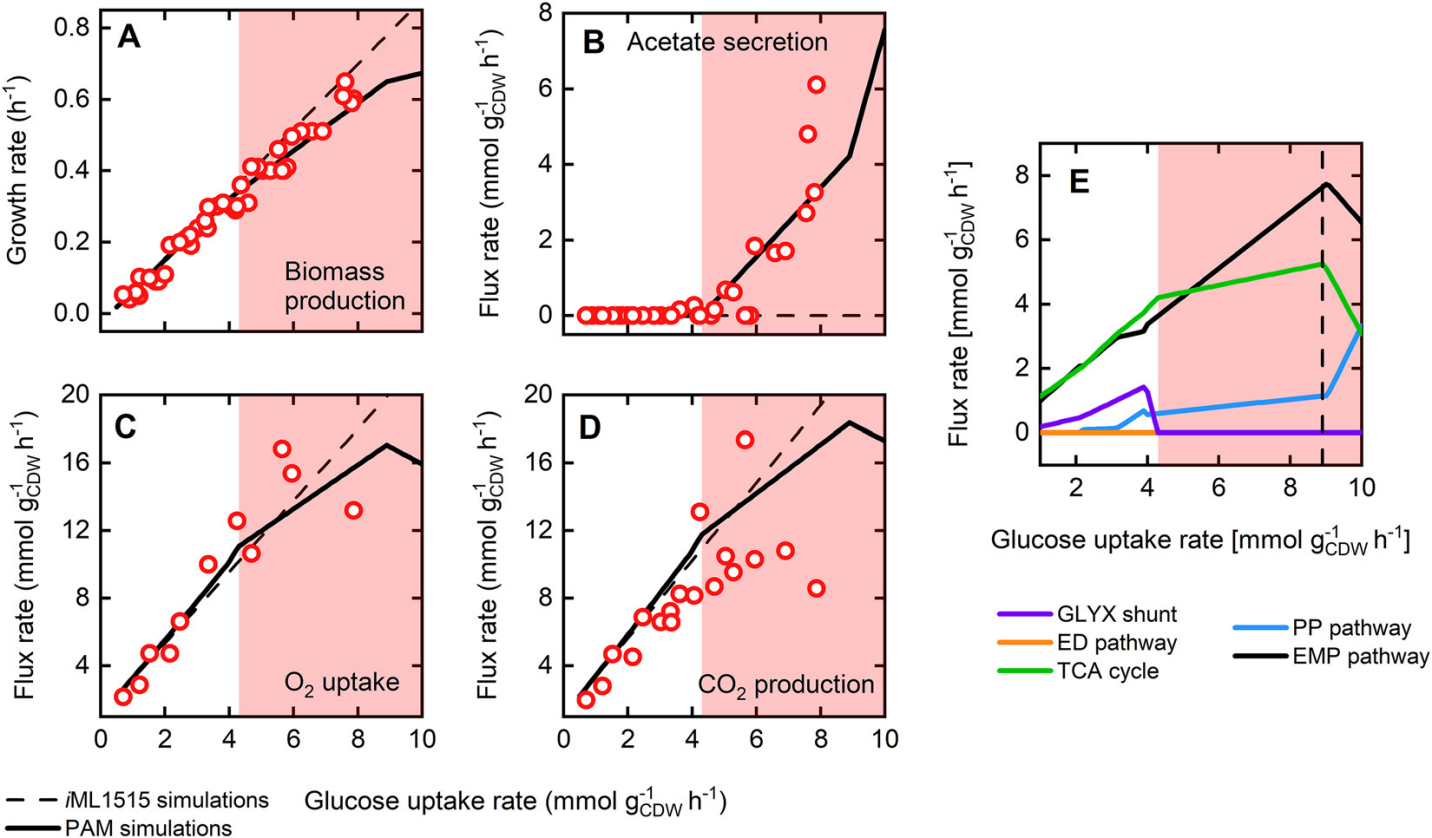
PAM predictions for E. coli phenotypes for a range of physiologically relevant glucose uptake rates in comparison to data from the literature. (A to D) Predictions (black lines) and experimental data (41, 52–56, 58, 59) (red dots) of (A) growth, (B) acetate secretion, (C) oxygen uptake, and (D) carbon dioxide production rates are compared. Simulation results using the original stoichiometric *i*ML1515 model are additionally shown (dashed lines). (E) Simulated fluxes through central metabolic pathways. The dashed line marks the maximum glucose uptake rate of 8.9 mmol ${\text{g}}_{\text{cdw}}^{-1}$ h^−1^, where the unused enzyme concentration *ϕ*_UE_ was defined to be zero. In all panels, the red shaded area marks the occurrence of fermentation resulting in the secretion of acetate.

To benchmark the PAM’s predictive capabilities, we simulated the wild-type phenotypic behavior on glucose minimal medium and compared the results to extensive literature data (52–59). The maximum glucose uptake rate, a parameter for the unused enzyme sector, was set to 8.9 mmol ${\text{g}}_{\text{cdw}}^{-1}$ h^−1^, which supported an observed maximal growth rate of 0.65 h^−1^ (52). The simulated data derived with this constraint is in good agreement with experimentally observed phenotypes for a range of carbon-limited conditions and depicts significant improvements compared to the purely stoichiometric *i*ML1515 model (Fig. 3). In particular, the acetate secretion trend correctly mirrors the metabolic overflow characteristics of E. coli above glucose uptake rates of 4.3 mmol ${\text{g}}_{\text{cdw}}^{-1}$ h^−1^ (Fig. 3B). Despite reasonable projections of growth, acetate secretion, and oxygen uptake rates, the PAM as well as the *i*ML1515 model overestimate carbon dioxide secretion rates, pointing to potential inconsistencies in the biomass synthesis equation used in both models.

The phenotypes simulated by the PAM are also mirrored in the fluxes through central metabolic pathways as shown in Fig. 3E. The distribution of the carbon flux among the metabolic pathways qualitatively follows previous findings (53). Under carbon-limited and fully respiratory conditions, flux rates through the Embden-Meyerhof-Parnas (EMP) pathway, the tricarboxylic acid (TCA) cycle, the pentose phosphate (PP) pathway, and the glyoxylate shunt (GLYXS) scale linearly with the glucose uptake rate. The GLYXS activity reflects the need for anaplerosis to compensate for the drainage of TCA cycle intermediates under slow growth conditions, which has been experimentally demonstrated (60).

In the vicinity of the turning point from a fully respiratory to partially fermentative metabolism, the activity of the GLYXS diminishes completely. Beyond glucose uptake rates of 4.3 mmol ${\text{g}}_{\text{cdw}}^{-1}$ h^−1^, limitations in the global protein household impede exclusive ATP production via respiration. As a consequence, the carbon flux is partly diverted from the NADH-yielding, and thus respiration-fueling, TCA cycle toward acetate. Simultaneously, the split between the EMP and PP pathways starts to increase with elevated glucose uptake rates in favor of glycolysis. While the NADPH supply through the PP pathway follows the growth-rate-dependent demand for biomass synthesis, the glycolytic flux (EMP) is accelerated to compensate for the carbon loss toward acetate.

The PAM’s stoichiometric and protein allocation constraints support feasible growth states beyond the assumed maximum glucose uptake rate (Fig. 3). However, the increase in growth rate is at the expense of biomass yield, which becomes evident by a drastic decrease of the flux through the EMP pathway and the TCA cycle and a progressively increasing acetate secretion rate. These simulation results do not necessarily indicate an underestimated maximum glucose uptake rate since PAM parameterization stays feasible even for maximum glucose uptake rates far beyond physiologically relevant values (data not shown). In fact, this suggests that the main metabolically limiting factor for E. coli is neither stoichiometry nor protein allocation. As we will show in the next section, maximal growth rates may be attributed to a limited, maximum molar protein synthesis rate, thus indicating transcriptional restrictions as the determining growth-limiting factor.

### Computing maximum substrate uptake rates.

Since glucose is the preferred carbon source of E. coli, its metabolism and regulation are adapted for an effective glucose utilization, which led us to assume that there are no unused enzymes under glucose-excess conditions. However, it is reasonable to assume that such a state of adaption does not hold for alternative carbon sources. Thus, experimentally observed substrate uptake rates may not reflect growth states with (near-)zero unused enzymes. To enable the PAM to simulate growth on alternative carbon sources without the need for cultivation data, we approximated maximum substrate uptake rates assuming a maximum total protein synthesis rate *N*_P,max_. *N*_P,max_ represents a global cellular constraint for protein biosynthesis, applicable for any substrate. It manifests at the maximum substrate uptake rate and therefore at maximum growth at which the unused enzyme sector approaches zero. In the PAM, *N*_P,max_ represents the sum of molar synthesis rates of proteins from the active enzymes and the translational sectors.

Based on the PAM and phenotypic data of E. coli grown on several substrates (61), we found that an *N*_P,max_ of 2.04 μmol ${\text{g}}_{\text{cdw}}^{-1}$ h^−1^ defines maximum substrate uptake rates (Table S1). For glucose as the sole carbon source, an uptake rate of 9.82 mmol ${\text{g}}_{\text{cdw}}^{-1}$ h^−1^ is predicted accordingly. Interestingly, experimental values suggest that the maximum growth rate on acetate is approached for an uptake rate of 19.6 mmol ${\text{g}}_{\text{cdw}}^{-1}$ h^−1^ and a protein synthesis rate below *N*_P,max_. This phenomenon indicates that protein allocation limits the maximum growth on acetate. Acetate is metabolized through the TCA cycle and the GLYXS (61). Energy-yielding routes other than respiration are biochemically impossible since substrate phosphorylation via the protein-efficient fermentation pathway results in acetate formation and, hence, a metabolic cycle with zero net carbon uptake. As a consequence, ATP generation solely relies on protein-costly respiration. We assume that the allocation of protein to the respiratory pathway is ultimately limited by the total protein concentration *ϕ*_P,c_. Thus, ATP supply and cofactor regeneration become the limiting factors before the maximum molar protein synthesis capability is reached.

10.1128/mSystems.00625-20.10TABLE S1*In silico* determined maximum substrate uptake rates according to a maximally allowable total protein synthesis rate *N*_P_ of 2.04 μmol ${\text{g}}_{\text{cdw}}^{-1}$ h^−1^. Since fully anaerobic conditions are infeasible for the PAM and also the original *i*ML1515 model, minimal oxygen uptake rates of 1.5 mmol ${\text{g}}_{\text{cdw}}^{-1}$ h^−1^ and 3.5 mmol ${\text{g}}_{\text{cdw}}^{-1}$ h^−1^ were used to simulate microaerobic growth on glucose and xylose, respectively. Download Table S1, DOCX file, 0.02 MB.Copyright © 2021 Alter et al.2021Alter et al.https://creativecommons.org/licenses/by/4.0/This content is distributed under the terms of the Creative Commons Attribution 4.0 International license.

**FIG 4:**
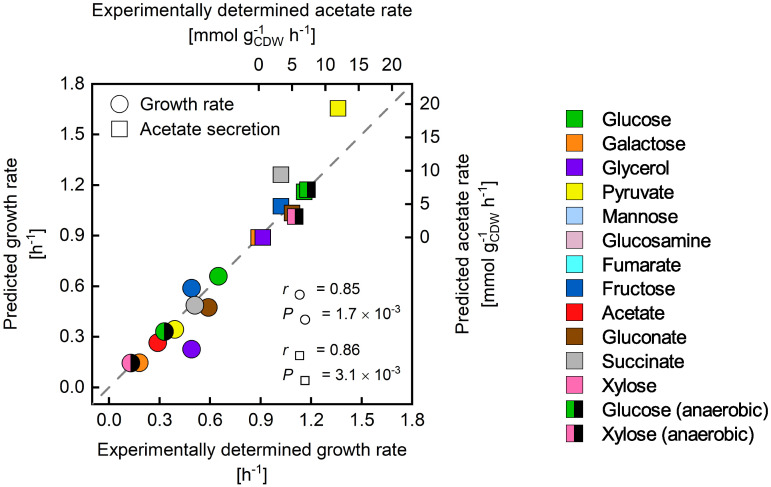
Comparison of experimentally determined (61) and predicted growth and acetate secretion rates on different, single carbon sources. Substrate uptake rates were constrained according to the reported values. Maximum substrate uptake rates were approximated according to a maximum total protein synthesis rate *N*_P,max_ (Table S1). The goodness of the correlations between simulations and experiments for growth and acetate secretion rates were determined using the Pearson correlation coefficient *r* and the corresponding *P* value.

By parameterizing the unused enzyme sector with the substrate-specific maximum substrate uptake rates, PAM simulations of phenotypes of E. coli grown on alternative carbon sources showed a good correlation with experimentally observed data (Fig. 4). The overestimation of growth and acetate secretion rates at the determined maximum substrate uptake rates (Fig. S1) indicates an unused potential to adapt E. coli to these alternative carbon sources. This assumption is, for instance, supported by a study by Fong et al. (62), in which the glycerol uptake rate was evolved to around 15 mmol ${\text{g}}_{\text{cdw}}^{-1}$ h^−1^, which is close to the PAM’s prediction. Interestingly, the shift from glucose to glycerol at the beginning of the evolutionary adaption of E. coli caused immediate, significant transcriptional responses. Regulatory mechanisms appeared to significantly affect the expression of genes associated with catabolism, such as *cra* and *crp*, presumably to nonspecifically scavenge and prepare for alternate substrates. During the adaptive evolution process, metabolism was focused on the available substrate, glycerol, and the basal transcriptional state was approached again. Thus, optimal glycerol uptake and utilization were mediated by changes in transcriptional regulation resulting in the adaption of catabolism to this sole carbon source. Moreover, flagellar and motility gene expression decreased during the adaptation, probably increasing the protein availability for growth-related processes.

10.1128/mSystems.00625-20.2 FIG S1 Comparison between experimentally determined and predicted maximum substrate uptake rates, as well as the corresponding growth and acetate secretion rates using several substrates as sole carbon sources. Maximum substrate uptake rates were approximated according to a maximum total protein synthesis rate. Download FIG S1, EPS file, 0.3 MB. Copyright © 2021 Alter et al. 2021 Alter et al. https://creativecommons.org/licenses/by/4.0/ This content is distributed under the terms of the Creative Commons Attribution 4.0 International license.

### Prediction of E. coli flux distributions.

**FIG 5:**
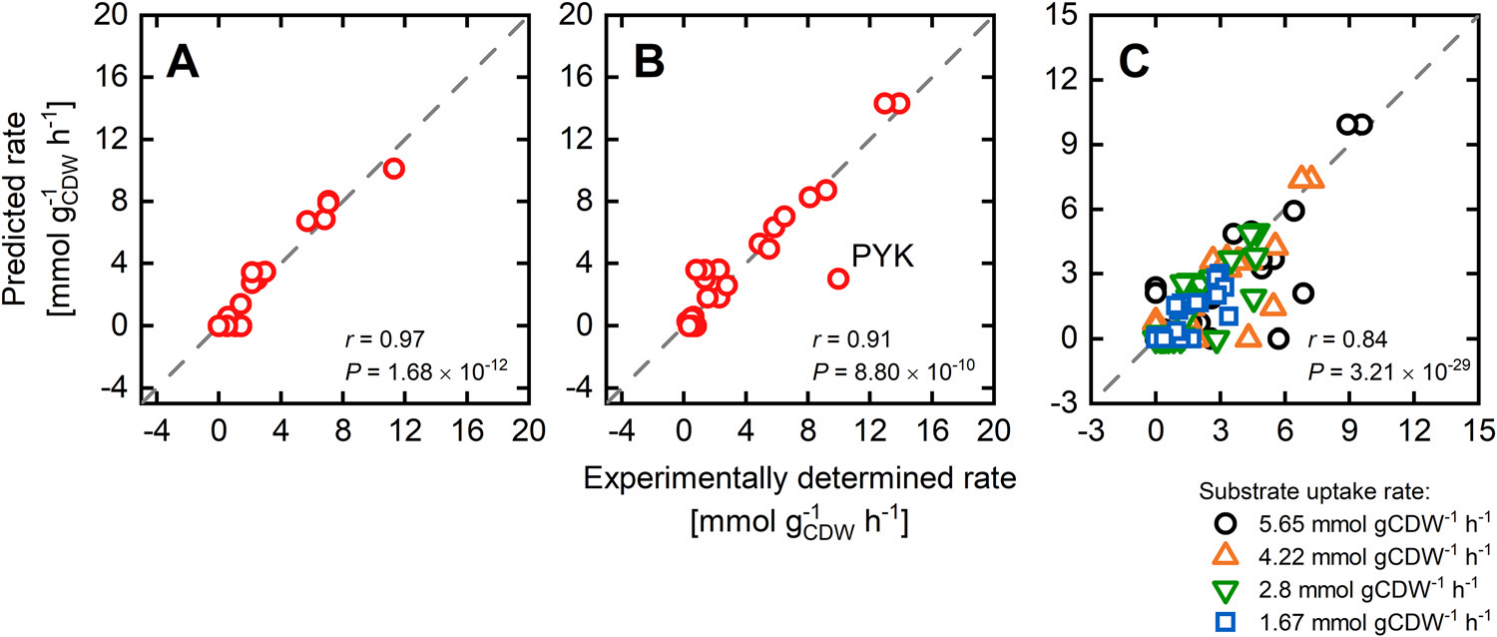
PAM predictions of intracellular fluxes of the central carbon metabolism of E. coli grown on a glucose minimal medium. The glucose uptake rate was constrained with experimentally determined values. The unused enzymes sector was parameterized according to the computationally determined maximum value of 9.82 mmol ${\text{g}}_{\text{cdw}}^{-1}$ h^−1^ based on a maximum total protein synthesis rate *N*_P_ of 2.04 μmol ${\text{g}}_{\text{cdw}}^{-1}$ h^−1^. (A to C) The predictions are compared with experimental flux data from panels A (61), B (63), and C (53). The goodness of the correlations was computed based on the Pearson correlation coefficient *r* and the corresponding *P* value.

By exploiting the optimality principles of microbial growth, GEMs give quantitative insights into the intracellular flux distribution and pathway usage, based purely on stoichiometric constraints. For a particular environmental condition of interest, the prediction accuracy of the stoichiometric GEM generally scales with the amount and accuracy of experimental data introduced to the model in the form of flux constraints. With a minimum need for such data, i.e., the substrate uptake rate, the PAM allows for an accurate blueprint of the intracellular metabolic processes. A comparison with fluxomics data from multiple studies shows that flux distributions of the central carbon metabolism of E. coli grown on a minimal glucose medium are well predicted by the PAM, which is indicated by Pearson correlation coefficients up to 0.97 (Fig. 5). Here, the PAM was constrained with the measured glucose uptake rates, and the unused enzyme sector was parameterized according to the methodically determined maximum glucose uptake rate, which, in turn, was a direct model output (cf. the previous section). Thus, protein allocation and enzymatic constraints alone enhance constraint-based modeling as a metabolic prediction tool, making this modeling approach particularly useful under data scarcity. The relatively large discrepancies between simulated and experimental flux data for the pyruvate kinase (53, 63) (Fig. 5B and C) result from the neglect of the phosphotransferase system (PTS) for glucose uptake in the ^13^C metabolic flux analysis models. The PTS system transfers the phosphate group from phosphoenolpyruvate (PEP) to glucose during its import, thereby producing glucose-6-phosphate and pyruvate. The omission of this alternative uptake system necessitates an elevated pyruvate kinase flux to balance PEP. In traditional ^13^C-based metabolic flux analysis, glucose uptake via the PTS and the ABC transporter cannot be dissected, as they do not affect the ^13^C isotope patterns of metabolites. It is therefore common to incorporate only one pathway in these models.

We further used the methodically determined maximum substrate uptake rates and constrained the PAM with measured uptake rates to predict flux distributions for nonglucose carbon sources (Fig. S2). Here, the prediction capabilities were more diverse, resulting in high correlations for acetate, galactose, and succinate (*r* > 0.92) but intermediate to weak predictions, e.g., for fructose or gluconate (*r* > 0.65). In the case of gluconate consumption, the Entner-Doudoroff (ED) pathway was experimentally observed to be the main catabolic route, whereas the simulated carbon flux was exclusively channeled toward pyruvate through the pentose phosphate (PP) pathway. The flux split between the PP and ED pathways is highly sensitive to the ratio in the protein demands of both pathways (data not shown). Consequently, inconsistencies in the applied *k*_cat_ values, particularly of backward reactions, but also the substrate-dependent differences in the biomass compositions (13) may cause these observed discrepancies. Further in-depth investigations are needed to validate one or the other assumption.

10.1128/mSystems.00625-20.3FIG S2Comparison of predicted growth and acetate secretion rates of GMS with experimentally determined values taken from Long et al. (70) and Fong et al. (72). Predictions were made applying the PAM and constraining the upper bound of the glucose uptake rate to the methodically determined maximum value of 9.82 mmol ${\text{g}}_{\text{cdw}}^{-1}$ h^−1^. Download FIG S2, EPS file, 0.3 MB.Copyright © 2021 Alter et al.2021Alter et al.https://creativecommons.org/licenses/by/4.0/This content is distributed under the terms of the Creative Commons Attribution 4.0 International license.

### PAM explains the growth defect upon heterologous protein expression.

Enzyme overproduction or the expression of heterologous genes is a common strategy in many biotechnological disciplines. General purposes are introducing novel cellular functionalities, flux enforcement through a specific pathway, or investigation on cellular processes via reporter proteins. In any case, the introduced pull of proteins from the limited native protein household and the resulting metabolic burden inevitably cause a growth defect.

**FIG 6:**
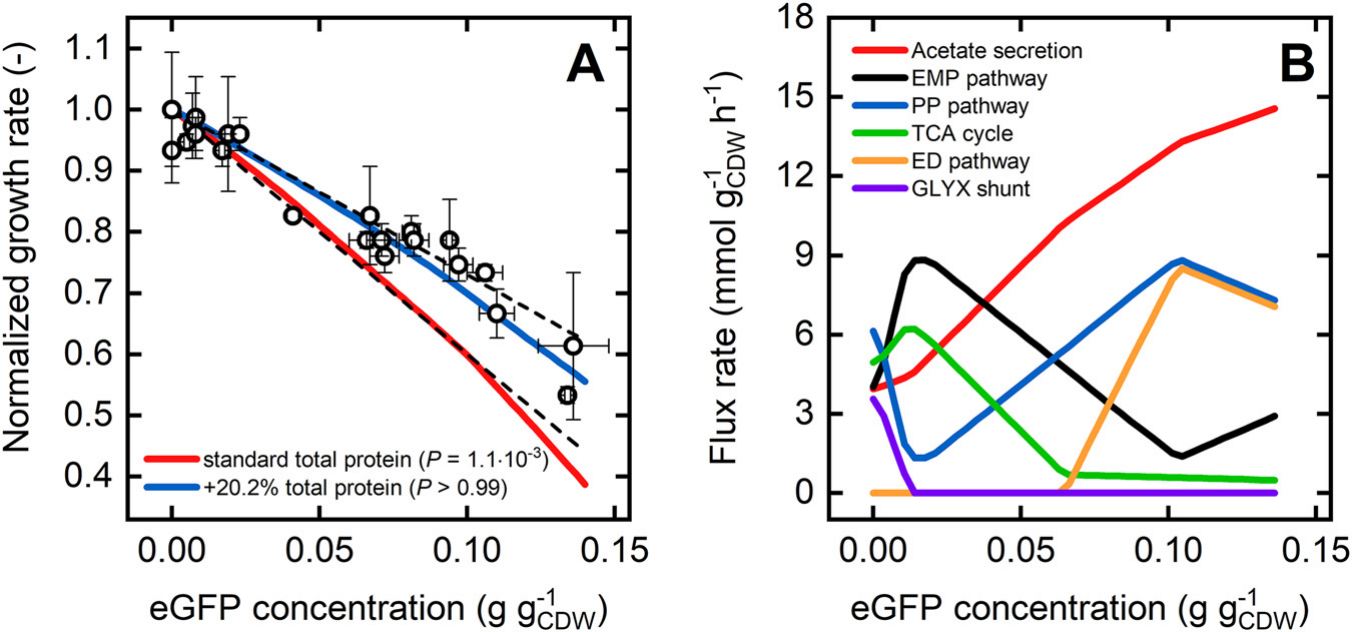
(A) Simulated growth rates relative to the maximum growth rate are shown for a range of intracellular eGFP concentrations using the PAM with a standard (red line) and increased (blue line) total condition-dependent protein concentration *ϕ*_P,c_ (0.26 g ${\text{g}}_{\text{cdw}}^{-1}$ and 0.31 g ${\text{g}}_{\text{cdw}}^{-1}$, respectively). Experimental data are taken from reference 64. *P* values are derived from Student’s t-test and indicate how well model predictions explain the experimental data. The dashed lines illustrate the predicted range of values based on theoretical considerations by Bienick et al. (64). (B) Simulated fluxes through central metabolic pathways are shown for a range of intracellular eGFP concentrations and a *ϕ*_P,c_ of 0.31 g ${\text{g}}_{\text{cdw}}^{-1}$.

To investigate and evaluate the response of the PAM to such an induced protein demand, we simulated growth for a range of expression levels of an enhanced green fluorescent protein (eGFP) and compared relative growth rates with experimental data from Bienick et al. (64). For the standard concentration of total condition-dependent protein *ϕ*_P,c_ of 0.26 g ${\text{g}}_{\text{cdw}}^{-1}$, the simulated relative growth defect is much more pronounced than to experimentally observed growth for intracellular eGFP concentrations beyond 0.03 g ${\text{g}}_{\text{cdw}}^{-1}$ (Fig. 6A). The use of an E. coli TUNER strain, a derivative of the genome-reduced BL21 strain optimized for protein expression, in this study, explains this discrepancy. In this strain, the protein requirements for the growth rate-independent housekeeping sector are reduced, and more resources are available for the metabolic, condition-dependent protein sectors, which can be reflected in the PAM by increasing the total protein concentration *ϕ*_P,c_. Accordingly, increasing the protein availability by expanding *ϕ*_P,c_ about 20% attenuates the detrimental effects of eGFP expression on growth and results in an excellent reproduction of experimentally observed phenotypes (Fig. 6A).

Interestingly, the relation between eGFP expression strength and growth predicted by the PAM is nonlinear, which contrasts with a previous theoretical postulation (64). The nonlinearity arises from a combined effect of a protein drain from the translational and the active metabolic enzyme sectors. The enforced protein drain toward eGFP causes a decline in the ribosome concentration, resulting in a reduced translation rate. Under the assumption of a constant total protein concentration, the protein allocation to the metabolic sectors producing biomass precursors, amino acids, and energy decreases, which, in a vicious cycle, further decelerates the protein production rate and growth. This intracellular tug-of-war for proteins is intrinsically manifested in the PAM, leading to outperformance in predicting protein overexpression phenotypes over too simplified coarse-grained models.

The PAM also discloses relevant effects of the protein drain on the pathway flux level. For an increasing eGFP expression strength and the accompanied protein deficiency, central carbon fluxes are progressively diverted to fermentation pathways (acetate secretion) and eventually to the ED pathway. Both routes are more protein efficient but yield fewer energy equivalents per substrate molecule than respiration or the EMP pathway (39, 65, 66). The PAM confirms this diversion of ATP generation from respiratory to fermentation pathways. The proportion of ATP generated by respiration, more precisely by the ATP synthase, in the total cellular ATP generation rate significantly decreases with an increasing eGFP expression (Fig. S3). Thus, the excelled protein burden enforces a shift toward protein-efficient, substrate-level phosphorylation in E. coli.

10.1128/mSystems.00625-20.4FIG S3Simulated ratio of ATP generated via respiration and the total ATP generation rate for a range of eGFP concentrations. The flux through the ATP synthase was used as the respiratory ATP generation rate. Growth rates relative to the growth rate at zero eGFP expression are additionally shown. The presented data are based on growth-optimal solutions of the PAM applying a total protein mass concentration of 0.31 g ${\text{g}}_{\text{cdw}}^{-1}$ and a maximum glucose uptake rate of 9.81 mmol ${\text{g}}_{\text{cdw}}^{-1}$ h^−1^. Download FIG S3, EPS file, 0.1 MB.Copyright © 2021 Alter et al.2021Alter et al.https://creativecommons.org/licenses/by/4.0/This content is distributed under the terms of the Creative Commons Attribution 4.0 International license.

### Limitations in the protein allocation of single enzymes lead to gene deletion mutant phenotypes.

Alongside the over- and heterologous expression of genes, rearrangement of metabolic networks and flux distributions by gene deletions is a core instrument in metabolic engineering. In recent years, many computational strain design methods have been developed to accelerate and rationalize the engineering of microbial cell factories. However, in contrast to the vast number of model-driven strain design and optimization methods (67), constraint-based methods have often proven unreliable in predicting phenotypes of gene deletion mutant strains (GMSs). While GMSs have been shown to evolve toward FBA-predicted phenotypes (68), observed growth defects and intracellular fluxes of nonevolved GMSs cannot be explained by stoichiometry and a cellular growth objective alone (69–71).

**FIG 7:**
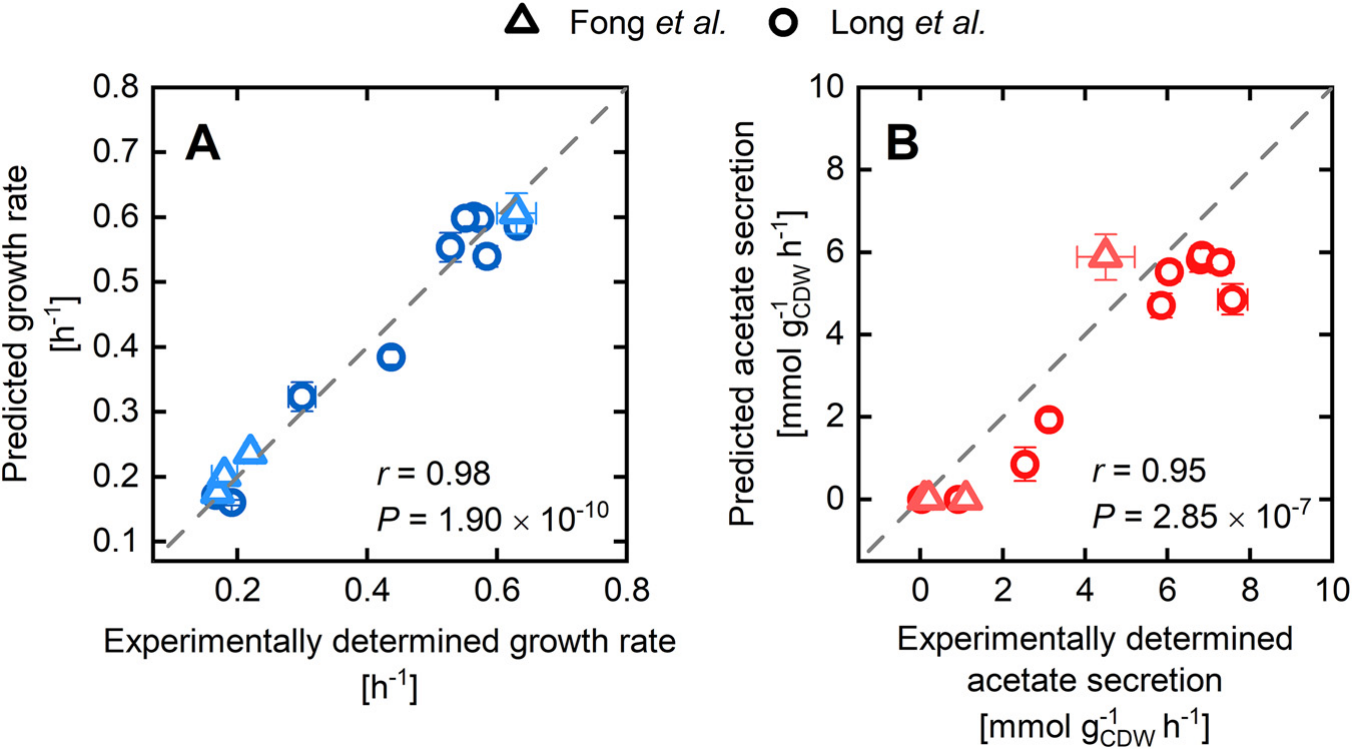
(A and B) Comparison of predicted growth (A) and acetate secretion rates (B) of gene-deletion mutant strains with experimentally determined values taken from references 70 (circles) and 72 (triangles). Predictions were made applying the PAM and constraining the glucose uptake rates to observed values.

First, we tested the impact of 10 single-gene deletions on the PAM’s FBA results by parameterizing the unused enzyme sector with the methodically determined maximum glucose uptake rate of 9.82 mmol ${\text{g}}_{\text{cdw}}^{-1}$ h^−1^. The calculated phenotypes did not significantly differ from the unperturbed wild-type solutions and, hence, did not compare to experimental data (70, 72) (Fig. S4). Only after constraining the glucose uptake rate to the measured values did the simulated growth and acetate secretion rates correlate well with the experiments (Fig. 7). Furthermore, a blind test in the form of FBA simulations augmented with *in vivo* glucose uptake rates of the GMS, but with an *intact* target gene, yielded the same phenotypes (Fig. S5). These results led us to conclude that the main driver for the observed growth defects of GMSs is a naturally orchestrated catabolite uptake repression induced by the respective network perturbations. A gene disruption resulting in a growth phenotype implicates a decrease in the levels of one or more amino acid precursors since tight proteome coordination (4) hampers enzyme allocation toward alternative precursor synthesis routes. According to the coarse-grained model of You et al. (4), which considers the general mechanism of rRNA transcription (73), low amino acid levels stall ribosomal protein synthesis via ppGpp. Moreover, low precursor levels, such as oxaloacetate or α-ketoglutarate, lead to an increased cAMP synthesis and elevated CRP-cAMP levels, which foster the expression of catabolic enzymes for alternative substrates adding to growth reduction. Elevated cAMP concentrations, which were experimentally observed in multiple GMSs (74), indicate increased CRP-cAMP activities and support this view on gene deletion triggering the integral feedback of metabolic control. Therefore, we argue that the degree of metabolic rewiring in GMSs necessary to overcome growth defects is often low. Indeed, restoration of growth can be achieved by evolutionary adaption processes that alter these regulatory patterns (74).

10.1128/mSystems.00625-20.5FIG S4Comparison of predicted growth and acetate secretion rates of GMS with experimentally determined values taken from Long et al. (70) and Fong et al. (72). Predictions were made applying the PAM and constraining the upper bound of the glucose uptake rate to the methodically determined maximum value of 9.82 mmol ${\text{g}}_{\text{cdw}}^{-1}$ h^−1^. Download FIG S4, EPS file, 0.2 MB.Copyright © 2021 Alter et al.2021Alter et al.https://creativecommons.org/licenses/by/4.0/This content is distributed under the terms of the Creative Commons Attribution 4.0 International license.

10.1128/mSystems.00625-20.6 FIG S5 Comparison of phenotypic predictions between the wild-type and gene-deletion mutant model. Predictions were made applying the PAM and constraining the upper bound of the glucose uptake rate to the observed values. Download FIG S5, EPS file, 0.2 MB. Copyright © 2021 Alter et al. 2021 Alter et al. https://creativecommons.org/licenses/by/4.0/ This content is distributed under the terms of the Creative Commons Attribution 4.0 International license.

The assumption that a regulatory substrate uptake inhibition shapes GMS’ phenotypes raised the question if the PAM can quantitatively predict substrate uptake modes. To tackle this question, we recalled computational frameworks such as minimization of metabolic adjustment (MOMA) (75), regulatory on/off minimization (ROOM) (76), and relative change (RELATCH) (69), which determine the metabolic impact of gene knockout strains. The common principle behind all three methods is the minimization of the metabolic response to genetic perturbations due to an unchanged regulatory system that forces the GMS’ flux distribution toward the original steady state. In the context of protein allocation, the minimal response principle can be translated as follows: upon a network perturbation, a GMS establishes a substrate uptake rate so that increases in protein allocation toward single enzymatic reactions are minimal compared to genetically unperturbed strains. The cellular objective is to allow for maximum metabolic activity in the face of knockout-induced flux rerouting and a hampered reallocation of protein due to a strict (wild-type) regulatory regime.

We applied the minimal response principle to the PAM for mutant phenotype predictions, and we simulated growth optimal flux distributions for a range of substrate uptake rates (cf. Materials and Methods for a detailed description). For each flux distribution, the difference in the synthesis rate Δ*N*_e_ between a reference, wild-type state at maximum growth and a mutant state is calculated for each enzyme within the PAM. The GMS’s maximum substrate uptake rate is determined from the flux distribution for which Δ*N*_e_ meets a defined upper bound $\Delta N_{\text{e}}^{\text{crit}}$ for at least one enzyme within that flux distribution. In doing so, we assign a certain flexibility to the overexpression capacity of single enzymes, which may be attributed to the activation of underutilized enzyme capacities. Moreover, if $\Delta N_{\text{e}}^{\text{crit}}$ is met for one enzyme, flux rerouting to circumvent the saturated enzyme reaction is impossible. Refusing substantial metabolic rewiring conforms to experimental observations that unevolved GMSs do generally not show a large amount of metabolic adjustment to the metabolic network perturbations (68, 75). The strict upper bound $\Delta N_{\text{e}}^{\text{crit}}$ reflects this metabolic rigidity on changes of enzyme synthesis rates in GMSs compared to the parental wild-type strain.

**FIG 8:**
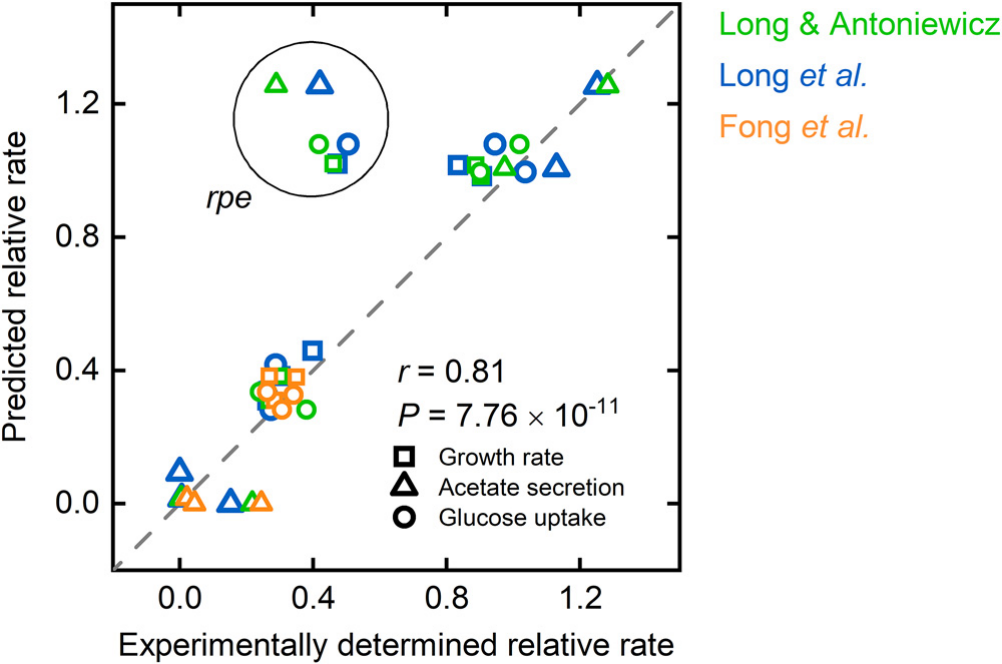
Comparison between simulated and experimentally determined phenotypes of GMSs without considering observed glucose uptake rates as model constraints. Predicted growth, glucose uptake, and acetate secretion rates are plotted against experimentally determined values taken from references 70 (blue), 71 (green), and 72 (orange). All values were normalized with corresponding wild-type data. Predictions were made applying the PAM and constraining the unused enzyme sector according to a maximum glucose uptake rate of 9.82 mmol ${\text{g}}_{\text{cdw}}^{-1}$ h^−1^. The maximum overexpression capacity of single enzymes $\Delta N_{\text{e}}^{\text{crit}}$ was set to 16 nmol ${\text{g}}_{\text{cdw}}^{-1}$ h^−1^.

Figure 8 shows the prediction results in comparison to experimentally determined phenotypic data (70–72) for an $\Delta N_{\text{e}}^{\text{crit}}$ of 16 nmol ${\text{g}}_{\text{cdw}}^{-1}$ h^−1^. $\Delta N_{\text{e}}^{\text{crit}}$ was derived from minimizing the discrepancy between simulated and experimentally observed phenotypes for various single-gene deletion mutants (cf. Materials and Methods for a detailed description). With one exception, there is a general agreement between predicted and experimentally determined phenotypes (Pearson correlation: *r* = 0.81, *P* = 7.76 × 10^–11^). By disregarding the Δ*rpe* deletion mutant, the Pearson correlation improves to *r* = 0.97 with a *P* value of 1.5 × 10^–23^. For the omitted outlier, simulations show a near-wild-type behavior, whereas growth is significantly reduced in the experiment. The *in vivo* observed growth defect of the Δ*rpe* GMS is peculiar since knockouts of enzymes adjacent to ribulose-phosphate 3-epimerase, such as *gnd*, do not show a similar phenotype. Even though the gene products of *gnd* and *rpe* catalyze consecutive reactions transforming 6-phospho-d-gluconate to d-xylulose 5-phosphate, an important intermediate in the PP pathway, deletion of *gnd* has a significantly smaller effect on growth, as does the deletion of *rpe*. Moreover, the experimentally determined phenotype of a Δ*rpe* GMS from reference 77 was similar to the wild-type E. coli strain and thus obscures a plausible judgment of the simulated phenotype.

**FIG 9:**
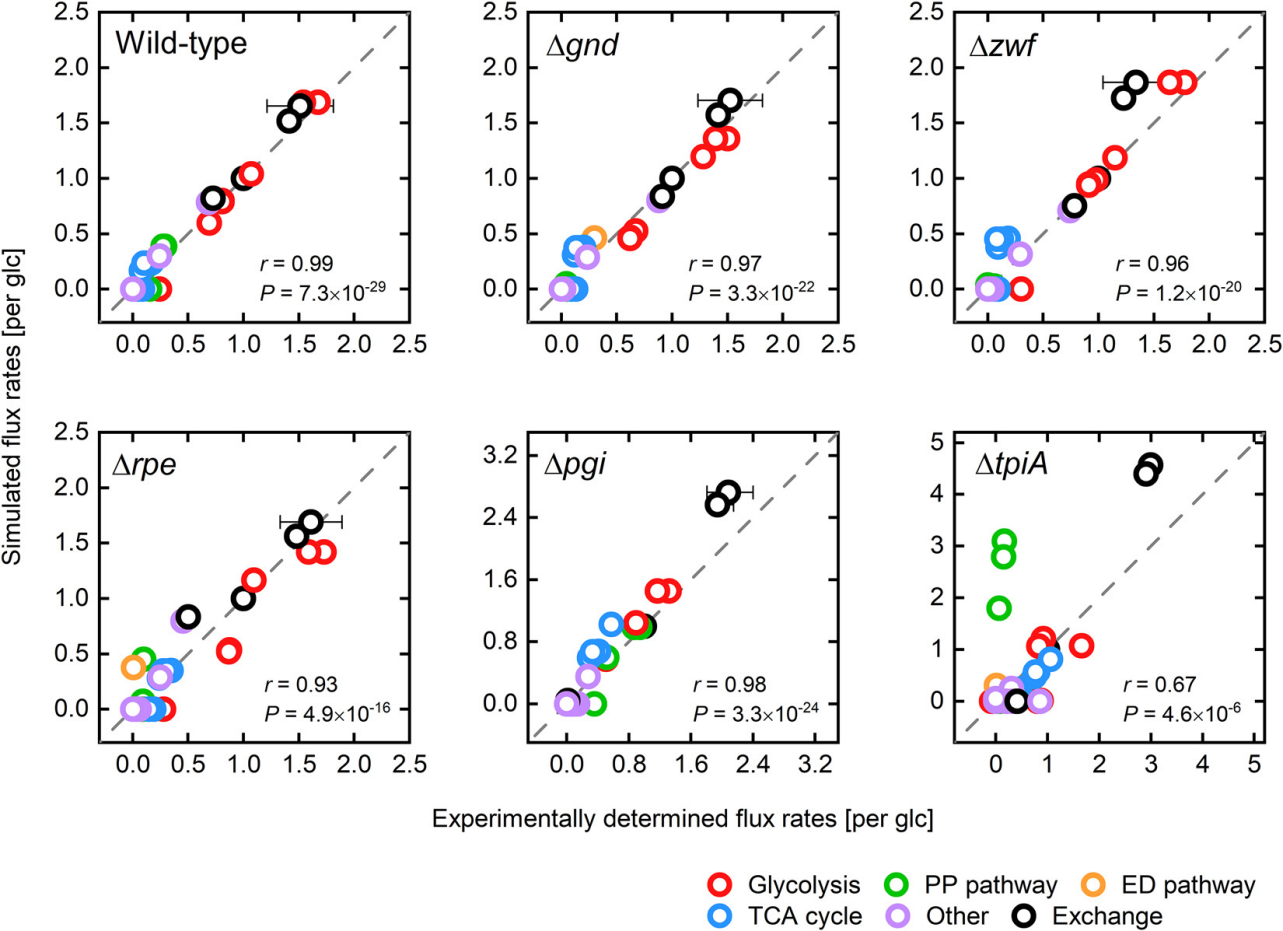
Comparison between experimentally determined and simulated flux distributions of GMSs without considering observed glucose uptake rates as model constraints. Predicted intracellular flux rates are plotted against experimentally determined values taken from Long and Antoniewicz (71). All data were normalized with corresponding glucose uptake rates. Predictions were made applying the PAM and constraining the unused enzyme sector according to a maximum glucose uptake rate of 9.82 mmol ${\text{g}}_{\text{cdw}}^{-1}$ h^−1^. The maximum overexpression capacity of single enzymes $\Delta N_{\text{e}}^{\text{crit}}$ was set to 16 nmol ${\text{g}}_{\text{cdw}}^{-1}$ h^−1^.

The considerable agreement between experimental and simulated phenotypic data also applies to intracellular flux data. We computed flux distributions for single-gene deletions that result in an experimentally proven inactivation of the corresponding reaction (i.e., we excluded single-gene deletions of isozymes) (71). The predicted flux responses correlated well with experimentally determined flux data (Fig. 9), noticeable on Pearson’s correlation coefficients of *r* > 0.93, for all tested single deletions, except for the Δ*tpiA* mutant (*r* = 0.67). For the Δ*tpiA* GMS, the experimentally observed rerouting of the glycolytic flux through the methylglyoxal pathway toward pyruvate (71, 78) contrasts with the PAM results, which suggests the activation of the ED pathway surpasses the blocked glycolysis. The simulated behavior can be traced back to an underestimated protein demand of the ED pathway, as predictions were improved after decreasing the *k*_cat_ values of the two central ED pathway reaction steps phosphogluconate dehydratase and 2-dehydro-3-deoxyphosphogluconate aldolase to 2.5% of the original values. After reevaluating the maximum glucose uptake rate, the simulations showed a diversion of the glycolytic flux into the methylglyoxal pathway and acetate secretion (Fig. S6). This selective adaption of turnover numbers, and thus of protein costs for the corresponding reactions, resulted in a significant convergence of simulated flux data toward measured values for the Δ*tpiA* and also the Δ*rpe* GMS (*r* of 0.90 and 0.99, respectively) (Fig. S7). However, at the same time, flux predictions for the Δ*gnd* mutant were deteriorated (*r* = 0.95). Here, flux was shifted from the ED pathway toward glycolysis, which contradicts experimental data. Nevertheless, the *k*_cat_ value adaption only slightly lessened the PAM’s overall predictive capabilities (Fig. S7) and thus generally points to the need for fine-tuning kinetic parameters to improve the flux split between pathways across different conditions.

10.1128/mSystems.00625-20.7FIG S6Phenotype predictions for GMS applying decreased *k*_cat_ values for the two central ED pathway steps compared to experimental observations. Experimentally determined growth, glucose uptake, and acetate secretion rates were taken from Long et al. (70) (blue), Long and Antoniewicz (71) (green), and Fong et al. (72) (red). All values were normalized with corresponding wild-type data. Predictions were made applying the protein allocation model by constraining the excess enzyme sector according to a maximum glucose uptake rate of 9.82 mmol ${\text{g}}_{\text{cdw}}^{-1}$ h^−1^ and by decreasing *k*_cat_ values for the two central ED pathway steps. Download FIG S6, EPS file, 0.1 MB.Copyright © 2021 Alter et al.2021Alter et al.https://creativecommons.org/licenses/by/4.0/This content is distributed under the terms of the Creative Commons Attribution 4.0 International license.

10.1128/mSystems.00625-20.8FIG S7Flux distribution predictions for GMS applying decreased *k*_cat_ values for the two central ED pathway steps compared to experimental observations. Experimentally determined intracellular flux rates were taken from Long and Antoniewicz (71). All values were normalized with corresponding wild-type data. Predictions were made applying the protein allocation model by constraining the excess enzyme sector according to a maximum glucose uptake rate of 9.82 mmol ${\text{g}}_{\text{cdw}}^{-1}$ h^−1^ and by decreasing *k*_cat_ values for the two central ED pathway steps. Download FIG S7, EPS file, 0.4 MB.Copyright © 2021 Alter et al.2021Alter et al.https://creativecommons.org/licenses/by/4.0/This content is distributed under the terms of the Creative Commons Attribution 4.0 International license.

## DISCUSSION

Proteins are the major molecular class in cells, and because they are the catalyst for global cellular functionalities, the importance of the mutual connection between microbial metabolic behaviors and protein allocation is broadly accepted (3, 5–7, 37–39). Based on existing techniques for implementing protein allocation and enzymatic constraints in constraint-based modeling (37, 40), we implemented a methodology to account for the total condition-dependent proteome in an E. coli GEM. Besides the integration of basic enzyme kinetics in the form of turnover numbers to model the concentrations of active enzymes, the resulting PAM considers simple, linear relations between microbial growth and the translational protein as well as the unused enzyme sector to describe 81% of the total protein mass concentration.

The PAM’s accurate predictions confirmed the prominent role of protein allocation in shaping microbial metabolism. Nevertheless, protein allocation itself appears to be regulated by biochemical limits. Genetic sequencing results of E. coli strains that have undergone extensive adaptive laboratory evolution (ALE) (79) suggest a causal link between transcription limitations and maximum cell proliferation rates. The adapted strains, all exhibiting a fitness increase of up to 1.6-fold, showed mutations in the *rpoB* or *rpoC* gene leading to single amino acid substitutions in the β/β′ subunit of the RNA polymerase. This subunit is part of the enzyme’s active center. Thus, the observed mutations globally affect transcription (79). We introduced the total molar synthesis rate of proteins as a proxy for the transcription capability of E. coli since the PAM does not represent any transcriptional processes. We found that maximum substrate uptake rates, and consequently maximum growth rates, are dictated by an ultimately limited total molar synthesis rate of condition-dependent proteins. Based on our study, we presume that a reduction of transcriptional limitations in evolved E. coli strains allows for an increased protein allocation toward the metabolically active enzymes and translational protein sector. A detailed proteomics study is necessary to uncover changing protein allocation principles in growth-optimized microbial strains and to test this hypothesis with the PAM.

By recognizing transcriptional limitations as hard constraints for the microbial metabolism and recalling that the presented PAM predictions represent growth optimal flux states, we deduce a cellular principle, which is supported by previous findings (39); particularly under substrate-limited conditions, E. coli regulates central cellular processes to maintain a Pareto-optimum between growth rate and the ability to adapt its metabolism flexibly to changing environmental conditions. The degree of flexibility is directly linked to the amount of allocated unused enzymes, which is hard-coded in the PAM. Interestingly, the cell retains a strictly substrate uptake-orientated allocation of protein to the unused enzyme sector even when protein becomes a metabolically limiting factor, apparent from the onset of overflow metabolism. This preservation of flexibility could allow for an evolutionary advantage in the original environmental niche. However, it may be a promising target for engineering E. coli or any microbe toward a high-performance cell factory.

Recently, systems metabolic engineering was emphasized as an integral part of the development and optimization of microbial cell factories (67, 80–83). Thus, we want to put the PAM forth to highlight the advantages of considering total condition-dependent proteome allocation for constraint-based modeling techniques that favor more accurate mutant strain predictions. We showed sound predictions of growth upon a range of overexpression levels of a nonenzymatic protein. The predictive capability for the overexpression of enzymes participating in metabolic reactions, e.g., in heterologously expressed pathways, still needs to be verified. However, the strict regulation of protein allocation, represented by the functional description of translational protein and unused enzyme sectors in the PAM, appeared to shape metabolic responses and is insensitive to genetic interferences. We also confirmed a rather inflexible protein allocation behavior for gene deletion mutant strains. Coherent prediction results were obtained by allowing only small divergences from a wild-type expression rate of single enzymes. Hence, in addition to the restrictions mediated by CRP-cAMP, we propose a link to transcriptional limitations, similar to our observation of maximum growth of wild-type strains mentioned above. This hypothesis is supported by ALE experiments in which adaption of single-gene deletion mutants frequently generated mutations affecting the regulation of global and pathway-specific transcription (74). These mutations possibly eliminate transcriptional hurdles and (partly) restore growth rates.

In summary, we want to stress the importance of protein allocation constraints in GEMs for the systematic constraint-based reconstruction and analysis (COBRA) and the design of microbial metabolism without having to sacrifice computational speed or applicability of established COBRA methods (84). However, the limited availability and credibility of enzymatic kinetic data, particularly of turnover numbers, still poses a significant obstacle in providing PAMs for any microorganism. Therefore, we join the call to establish a thorough kcatome as part of an accessible, genome-wide kinetome (85).

## MATERIALS AND METHODS

### Formulating the protein allocation model.

The protein allocation model (PAM) is based on the E. coli K-12 MG1655 GEM *i*ML1515 reconstruction (45). It includes linear representations of the protein mass concentrations of the active enzymes, the unused enzymes, and the translational protein sector as additional constraints. The sum of the protein mass concentrations of all sectors is kept constant according to Equation 1. The modeled protein sectors are described in detail in the following.

### The translational protein sector.

In agreement with previous studies, a linear correlation between the translational protein sector *ϕ*_T_ and the growth rate *μ* (5, 41) was implemented. Therefore, the inverse of the maximum ribosomal elongation rate *w*_T_ and a measure *ϕ*_T,0_ for an increasing overcapacity of ribosomes with a decreasing growth rate (43, 86) were used as the slope and intercept, respectively:

(2) $$\phi_{\text{T}}=\phi_{\text{T,0}}+w_{\text{T}}\;\mu$$

Both parameters *w*_T_ and *ϕ*_T,0_ were determined for E. coli by fitting Equation 2 to measured, cross-conditional concentration data of the translational protein sector (41) (Fig. 2B), resulting in values of 50.0 mg ${\text{g}}_{\text{cdw}}^{-1}$ (19% of the total protein mass concentration *ϕ*_P,c_ of 0.26 g ${\text{g}}_{\text{cdw}}^{-1}$) and 36.8 mg h ${\text{g}}_{\text{cdw}}^{-1}$ for *ϕ*_T,0_ and *w*_T_, respectively. The relative value for *ϕ*_T,0_ differs from the visual intercept in Fig. 2B, since protein mass concentrations in Fig. 2 were related to the total protein concentration of 0.32 g ${\text{g}}_{\text{cdw}}^{-1}$ measured by Schmidt et al. (41).

### The unused enzyme sector.

An evolutionary characteristic of microbes facing unforeseeable changes in environmental conditions is the synthesis of unused proteins. This protein hedging empowers the cell to quickly increase central carbon metabolism fluxes upon a sudden increase in substrate availability or to immediately catabolize a new carbon source (44). Enzyme overabundance also facilitates metabolic robustness against genetic perturbations (87). However, these protein reserves significantly reduce the growth rate, discussed in depth by O’Brien et al. (44) and Scott et al. (3). Protein expression and (over)allocation is generally coordinated by the cyclic AMP (cAMP)-dependent signaling pathway via the cAMP-activated global transcriptional receptor protein (CRP) (4, 88) (Fig. 1). The CRP-cAMP complex enhances the transcription of over 100 genes by attaching near or at their promoter regions, thereby mediating the binding of RNA polymerase for transcription initiation (89). The target genes are mainly associated with catabolism, and thus, CRP-cAMP aids in stimulating the carbon influx leading to the accumulation of precursors for amino acids and stimulation of protein synthesis. Amino acid precursors, such as oxaloacetate or α-ketoacids, inhibit the cAMP synthesis, thus lowering the CRP-cAMP level and closing the negative feedback loop between protein precursors and substrate uptake. We refer to You et al. (4) for respective insights into the cAMP signaling pathway. In this way, CRP-cAMP indirectly coordinates the global protein allocation, including the allocation of unused enzymes, even though the transcription of only a small fraction of genes is directly affected. Considering the cAMP-controlled signaling pathway as a blueprint for protein synthesis regulation, we modeled the unused enzyme sector as a negative linear function of the substrate uptake flux, which we mathematically expressed as

(3) $$\phi_{\text{UE}}=\phi_{\text{UE,0}}-w_{\text{UE}}\;\nu_{\text{s}}$$

where *ϕ*_UE,0_ is the unused enzyme concentration at zero substrate uptake, and *ν*_S_ is the substrate uptake rate. *w*_UE_ relates the decrease of the unused enzymes’ concentration to the increase in *ν*_S_. *ϕ*_UE,0_ was determined from ME model simulations of un- and underutilized enzymes (44), resulting in a value of 0.17 g ${\text{g}}_{\text{cdw}}^{-1}$ equivalent to 65% of the total protein mass concentration *ϕ*_P,c_. At zero substrate uptake rate (*ν*_S_ = 0), and therefore at zero growth, the sum of unused enzymes and translational protein *ϕ*_UE,0_ + *ϕ*_T,0_ is 0.22 g ${\text{g}}_{\text{cdw}}^{-1}$. Thus, near *ν*_S_ = 0 the PAM explains only a fraction (84%) of the total protein mass concentration *ϕ*_P,c_ which is, however, assumed to be constant under any given condition. We expect this discrepancy to be caused by deviations from an otherwise constant total protein household in severely starving cells or specific protein requirements during non-growth conditions. The fraction not explained by the PAM is compensated for by nonphysiological, random activation of metabolic fluxes when calculating growth-optimal solutions near substrate uptake rates of zero. Thus, the PAM may produce feasible solutions at low metabolic activity but hardly represent physiologically relevant flux states.

The slope *w*_UE_ is a measure of the increase in enzyme usage efficiency with increasing substrate uptake rate. It is individually assigned for each substrate under the assumption that the unused enzyme concentration is zero at the maximum substrate uptake rate. Thus, *w*_UE_ is calculated from

(4) $$w_{\text{UE}}=\frac{\phi_{\text{UE,0}}}{\nu_{\text{s,max}}}$$

The maximum substrate uptake rate $\nu_{\text{S,max}}$ needs to be provided as an observable but can be directly inferred from the PAM by assuming an upper limit in the transcriptional capacity, as shown in “Determination of Maximum Substrate Uptake Rates.”

### The active enzyme sector.

To account for the enzyme demand of metabolic fluxes, we integrated enzyme mass balances for all relevant metabolic reactions in the stoichiometric matrix of *i*ML1515 according to the GECKO framework (40), as explained in the following. The employed enzyme mass balance formulation (Equation 5) relates the flux *ν*_e_ to the minimally required concentration *ρ*_e_ of the enzyme catalyzing reaction e by the enzyme’s turnover number *k*_cat,e_. Since the PAM accounts for the total proteome by mass, we transformed molar to mass concentrations using the molar mass *M*_e_ of each enzyme in the GEM.

(5) $$\rho_{\text{e}}=\nu_{\text{e}}\frac{M_{\text{e}}}{k_{\text{cat}}{}_{\text{,e}}}$$

Eventually, the sum of the concentration of all *E* enzymes constitutes the metabolically active enzyme sector *ϕ*_AE_ and is expressed as

(6) $$\phi_{\text{AE}}=\sum_{\text{e}}^{E}\rho_{\text{e}}$$

Parameterization of the active enzyme sector is important to facilitate a meaningful relation between fluxes and enzyme concentrations. For the PAM, we determined *k*_cat_ values for 2,843 enzymes of the *i*ML1515 model from queries of the BRENDA (90) database following the protocol of Sánchez et al. (40).

Since simulated maximum growth rates were unreasonably low when applying the initial *k*_cat_ set, the *k*_cat_ data set was manually curated to allow for the computation of reasonable phenotypes (cf. Data Set S1, sheet 1 for the final data set). Therefore, enzymes were ranked according to the sensitivity of FBA-derived, maximal growth rates toward their *k*_cat_ values. Approximately 150 *k*_cat_ parameters that were found to be most influential on computed growth rates were reevaluated. For most of the underlying enzymes, no specific *k*_cat_ entry could be found in BRENDA, and *k*_cat_ values had been taken from close enzyme classes during the automated database queries. This *k*_cat_ approximation was corrected by manually querying SABIO-RK (91), UniProt (92), and primary literature for more sound values regarding growth optimality. If no specific data could be found for an enzyme, *k*_cat_ values from adjacent enzyme classes were manually selected and assigned to the PAM.

10.1128/mSystems.00625-20.1 DATA SET S1 **(**Sheet 1) Curated enzymatic data used to build the protein allocation model (data shown in Fig. S8). (Sheet 2) Transformation of experimentally determined protein mass data to dry cell weight-related units. (Sheet 3) Cell volumes calculated from growth rates based on the experimentally justified relation of Volkmer and Heinemann (95). (Sheet 4) Experimental data of growth, acetate secretion, oxygen uptake, and carbon dioxide production rates for E. coli for a range of glucose uptake (data shown in Fig. 3). (Sheet 5) Comparison between experimentally determined and predicted growth and acetate secretion rates using several substrates as the sole carbon (data shown in Fig. 4). (Sheet 6) Comparison between predictions and experimental data of intracellular fluxes of the central carbon metabolism of E. coli grown on a glucose minimal medium (data shown in Fig. 5). (Sheet 7) Comparison of predicted and experimentally determined intracellular fluxes of the central carbon metabolism of E. coli grown on several alternative carbon sources (data shown in Fig. S2). (Sheet 8) Experimentally determined, normalized growth rates for a range of eGFP expression strengths and respective eGFP concentrations in E. coli (data shown in Fig. 6). (Sheet 9) Comparison of predicted growth and acetate secretion rates of single-gene deletion E. coli mutants with experimentally determined values (data shown in Fig. 7). (Sheet 10) Comparison of predicted relative growth, glucose uptake, and acetate secretion rates of single-gene deletion E. coli mutants with experimentally determined values (data shown in Fig. 8). (Sheet 11) Comparison of predicted intracellular flux rates of single-gene deletion mutants with experimentally determined values (data shown in Fig. 9). (Sheet 12) Comparison between experimentally determined and predicted growth and acetate secretion rates using several substrates as the sole carbon sources (data shown in Fig. S1). (Sheet 13) Comparison of predicted growth and acetate secretion rates of single-gene deletion E. coli mutants with experimentally determined values (data shown in Fig. S4). (Sheet 14) Comparison of phenotypic predictions between wild-type and gene-deletion mutant model (data shown in Fig. S5). (Sheet 15) Comparison of predicted relative growth, glucose uptake, and acetate secretion rates of single-gene deletion E. coli mutants for adapted *k*_cat_ values of both ED pathway steps with experimentally determined values (data shown in Fig. S6). (Sheet 16) Comparison of predicted intracellular flux rates of single-gene deletion mutants for adapted *k*_cat_ values of both ED pathway steps with experimentally determined values (data shown in Fig. S7). (Sheet 17) Comparison between turnover numbers from the *in vitro* curated data set and a data set derived from a machine learning ensemble model published by Heckmann et al. (94) (data shown in Fig. S8). Download Data Set S1, XLSX file, 0.3 MB. Copyright © 2021 Alter et al. 2021 Alter et al. https://creativecommons.org/licenses/by/4.0/ This content is distributed under the terms of the Creative Commons Attribution 4.0 International license.

The manual curation effort transformed the primary *in vitro k*_cat_ estimates to effective (or apparent) *in vivo* turnover numbers *k*_app_ (48, 93), relating enzyme concentrations to metabolic fluxes under unlimited growth conditions. Recently, measurements of *k*_app,max_, the maximum *k*_app_ values across conditions, were extrapolated to genome scale using machine learning models informed with biochemical and enzymatic data (94). Interestingly, although the distribution of *k*_app,max_ values is significantly different from our curated set (Fig. S8), simulations using the PAM parameterized with either or both *k*_app,max_ sets yielded comparable phenotypes (data not shown). Thus, it appears that the ratio of *k*_cat_ values between different reactions or pathways is an essential factor for the predictive capability of PAMs in general.

10.1128/mSystems.00625-20.9 FIG S8 (A to C) Relative fraction (A), cumulative distribution (B), and direct comparison (C) of *k*_cat_ (*in vitro* curated) and *k*_app,max_ (machine learning [ML] model) values. *k*_cat_ values are curated data extracted from the BRENDA, SABIO-RK, and UniProt databases, whereas *k*_app,max_ values were taken from Heckmann et al. (94). The green circles in panel C highlight enzymes of the central carbon metabolism assigned to *i*ML1515 subsystems “Glycolysis/Gluconeogenisis,” “Oxidative Phosphorylation,” “Citric Acid Cycle,” “Pentose Phosphate Pathway,” and “Pyruvate Metabolism.” FIG S8, EPS file, 0.5 MB Copyright © 2021 Alter et al. 2021 Alter et al. https://creativecommons.org/licenses/by/4.0/ This content is distributed under the terms of the Creative Commons Attribution 4.0 International license.

### Processing of experimental proteomic data from literature.

The experimental proteomic data set generated by Schmidt et al. (41) was used in this study to parameterize the translational and the unused enzymes sector of the PAM. To fit the units of the data set to the model variables, the protein mass per cell measured by Schmidt was converted to dry cell weight-based concentration values:

(7) $$\phi_{\text{P}}=\frac{M_{\text{P}}}{V_{\mu }c_{\text{cdw}}}$$

Here, *ϕ*_P_ is the intracellular concentration (g ${\text{g}}_{\text{cdw}}^{-1}$), and *M*_P_ is the experimentally determined mass per cell of a protein or a protein sector *P*. *V_μ_* is the growth rate-dependent cellular volume, which was thoroughly determined by Volkmer and Heinemann (95). The cell dry weight (cdw) concentration per cell volume *c*_cdw_ was assumed to be 268.36 g_cdw_ liter^−1^ (50). Refer to Data Set S1, sheets 2 and 3 for the complete data set.

UniProt identifiers (92) and the COG classification (Clusters of Orthologous Groups) were used to match the proteins and protein sectors of the PAM to the proteomic data.

### Solving the protein allocation model problem.

All flux solutions and corresponding phenotypes in this work represent growth-optimal solutions of the following, classical FBA-problem amended with additional protein allocation and enzymatic constraints:

(8) $$\begin{array}{l} \underset{\bar{\nu }\in {\mathbb{R}}^{\left|N\right|},\bar{\rho }\in {\mathbb{R}}^{\left|E\right|}}{\max}\mu \\ s.t.\text{Classical FBA constraints}\\ S\bar{\nu }=\bar{0}\begin{array}{l} \nu_{\text{i}}^{\text{lb}}\leq \nu_{\text{i}}\leq \nu_{\text{i}}^{\text{ub}}\\ \nu_{\text{i}}\geq 0 \end{array}\}\forall \text{i}\in N\\ \text{Protein allocation constraints}\\ \begin{array}{l} \rho_{\text{e}}=\nu_{\text{e}}\frac{M_{\text{e}}}{k_{\text{cat}}{}_{\text{,e}}}\\ \rho_{\text{e}}\geq 0 \end{array}\}\forall \text{e}\in E\\ \phi_{\text{T}}=\phi_{\text{T,0}}+w_{\text{T}}\;\mu \\ \phi_{\text{UE}}=\phi_{\text{UE,0}}-w_{\text{UE}}\;\nu_{\text{s}}\\ \phi_{\text{P,c}}=\phi_{\text{T}}+\phi_{\text{UE}}+\sum_{\text{e}}^{E}\rho_{\text{e}} \end{array}$$

Here, *S* is the stoichiometric matrix of the original GEM, and *ν*_i_ is the flux variable of reaction i from the metabolic reaction pool *N*. Note that each reversible reaction in the original GEM is split into irreversible forward and backward reactions to only allow for positive flux values; hence, the lower and upper flux bounds $\nu_{\text{i}}^{\text{lb}}$ and $\nu_{\text{i}}^{\text{ub}}$ are equal to or greater than zero. The additional protein allocation and enzymatic constraints comprise the mass concentrations *ρ*_e_ for each considered enzyme or enzyme complex, as well as the mass concentrations of the ribosomal and unused enzyme sector *ϕ*_T_ and *ϕ*_UE_, respectively. All protein allocation constraints in Equation 8 were added to the stoichiometric matrix *S* of the GEM *i*ML1515 representing the E. coli K-12 MG1655 strain (45).

Each reaction e from pool *E*, comprising all reactions linked to one or more genes via a gene-protein-reaction (GPR) relation, is assigned to precisely one protein with a unique turnover number *k*_cat,e_ and molar mass *M*_e_. In case a reaction e is catalyzed by an enzyme complex (multiple genes are connected via logical AND operators in the GPR relation), the molar mass *M*_e_ is the sum of molar masses of the participating gene products. If two or more enzymes can catalyze the same reaction independently from each other (multiple genes are connected via logical OR operators in the GPR relation), the isozymes are merged into one hypothetical protein with a molar mass equal to the mean of the molar masses of the merged isozymes. Molar masses of enzymes were calculated as the sum of molar masses of the amino acids constituting the respective primary sequences reduced by the mass of one water molecule per peptide bond. The amino acid sequences were retrieved from the KEGG database (96) by queries with the GEM-inherent, KEGG-specific gene identifiers. The *k*_cat_ values for all enzymatic reactions in the model were derived as described in the previous section. The final set of curated *k*_cat_ values and molar masses of enzymes used throughout this study can be found in Data Set S1, sheet 1.

The translational and unused enzyme sectors *ϕ*_T_ and *ϕ*_UE_ are linearly related to the growth rate *μ* and the substrate uptake rate *ν*_s_, respectively. The linear equations of both sectors (Equation 8) are parameterized with condition-dependent proteomic data (41, 44) as discussed in the respective Materials and Methods sections and, except for the maximum substrate uptake rate *ν*_s_, parameters are maintained for any simulation in this work. Finally, the total mass concentration of condition-dependent protein *ϕ*_P,c_ is kept constant according to Equation 1 and comprises the sum of the translational sector *ϕ*_T_, unused enzyme sector *ϕ*_UE_, and active enzyme sector *ρ*_e_. An overview of the applied parameters is given in Table 2.

**TABLE 2:** Source and values of universal PAM parameters applied for all simulations in this study

Parameter	Symbol	Value	Source
Total protein concentration	*ϕ*_P,c_	258.0 mg ${\text{g}}_{\text{cdw}}^{-1}$	Derived from the sum of experimentally determined protein masses under different environmental conditions (41)
Intercept translational protein sector	*ϕ*_T,0_	49.9 mg ${\text{g}}_{\text{cdw}}^{-1}$	Derived from fitting and extrapolating proteomic data of translational protein (41)
Slope translational protein sector		36.8 mg h ${\text{g}}_{\text{cdw}}^{-1}$	Derived from fitting proteomic data of translational protein (41)
Intercept unused enzymes sector	*ϕ*_UE,0_	171.1 mg ${\text{g}}_{\text{cdw}}^{-1}$	Derived from fitting and extrapolating proteomic data of all proteins included in the *i*ML1515 metabolic model (41, 44)

### Determination of maximum substrate uptake rates.

By applying the PAM with parameterized protein sectors and minimal medium constraints, maximum uptake rates for single substrates were determined assuming a maximally allowable total protein synthesis rate *N*_P,max_. *N*_P_ represents the sum of molar synthesis rates of proteins from the active enzymes, unused enzymes, and the translational sector. At the maximum substrate uptake rate, and therefore at maximum growth, the unused enzyme sector is zero. Thus, the maximum total protein synthesis rate *N*_P,max_ is

(9) $$N_{\text{P,max}}=\mu \left(\frac{\phi_{\text{T}}}{M_{\text{R}}}+\overset{E}{\underset{\text{e}}{\sum }}\nu_{\text{e}}/k_{\text{cat}}{}_{\text{,e}}\right)$$

Here, *M*_R_ is the sum of the molar masses of all 21 ribosomal subunits of 3.5 × 10^5^ g mol^−1^, and *ν*_e_ is the fluxes of each enzymatically catalyzed reaction e.

We hypothesized that there is a unique maximum total protein synthesis rate *N*_P,max_ which determines maximum uptake rates for any given substrate. Knowledge of *N*_P,max_ would allow the determination of maximum substrate uptake rates and the parameterization of the unused enzyme sector (cf. Equations 3 and 4) solely based on model simulations. A correspondingly parameterized PAM should therefore be able to correctly predict phenotypes for substrate-limited conditions.

To test this hypothesis, we simulated *N*_P,max_ values for substrates considered by Gerosa et al. (61) using PAMs parameterized with a wide range of maximum substrate uptake rates. More specifically, the unused enzyme sector was parameterized at each of the tested maximum substrate uptake rates according to Equation 4. Growth rates were simulated by optimizing the parameterized PAMs for growth and constraining the substrate uptake rate to the experimentally determined value. These simulated growth rates were then compared with the observed values by computing the absolute difference. At an *N*_P,max_ of 2.04 μmol ${\text{g}}_{\text{cdw}}^{-1}$ h^−1^, the sum of absolute differences between simulated and measured growth rates was minimal. For each of the considered substrates, maximum uptake rates (parameter of the PAM) leading to *N*_P,max_ by applying the experimentally observed substrate uptake rate (constraint) were extracted and are shown in Table S1.

### eGFP overexpression.

Expression of eGFP was simulated by introducing an additional column to the stoichiometric matrix of the PAM representing a protein with a mass of 2.8 × 10^4^ g mol^−1^. Thus, the expression strength of eGFP (g h^−1^ ${\text{g}}_{\text{cdw}}^{-1}$) is controlled by the respective model variable describing the protein’s intracellular concentration and the growth rate. To identify the total protein concentration that represents the elevated protein availability in the E. coli TUNER strain used by Bienick et al. (64), the correlation between eGFP concentrations and growth rates relative to the wild-type was computed with the PAM for a range of total protein concentration values *ϕ*_P,c_. For each *ϕ*_P,c_, the summed difference between simulated and measured relative growth rates was calculated as a measure for the agreement between simulation and experiment. Student’s t-test was applied to determine the *ϕ*_P,c_ yielding the best possible correlation between measurements and simulations.

### Phenotype determination of gene deletion mutants.

The deletion of a gene was simulated by identifying all reactions connected to this gene via the model-inherent GPR rules and setting the respective upper bounds to zero. To predict maximum substrate uptake rates of GMSs, enzyme synthesis rates were calculated from growth-optimal flux distributions for a wide range of substrate uptake rates. The synthesis rate *N*_e_ of an enzyme e was computed from flux distributions by

(10) $$N_{\text{e}}=\rho_{\text{e}}\cdot \mu$$

where *μ* and *ρ*_e_ are the growth rate and the molar concentration of enzyme e, both being optimization variables to the PAM.

For each tested substrate uptake rate, the maximum difference in the computed enzyme synthesis rate $\Delta N_{\text{e}}^{\text{max}}$ between the GMS and a reference state representing a wild-type strain grown under substrate-unlimited conditions was identified among all modeled enzymes. By scanning $\Delta N_{\text{e}}^{\text{max}}$ values from high to low substrate uptake rates, the maximum substrate uptake rate for a GMS is found as soon as $\Delta N_{\text{e}}^{\text{max}}$ meets a critical value $\Delta N_{\text{e}}^{\text{crit}}$ (also termed maximum overexpression capacity). Thus, the corresponding metabolic mode supports a maximally achievable growth rate under a limited flexibility to change or reallocate protein among metabolic pathways and their single reactions. We assumed the level of restriction in flexibility $\Delta N_{\text{e}}^{\text{crit}}$ to be the same in any GMS according to phenotypic data of GMS from Long et al. (70), Long and Antoniewicz (71), and Fong et al. (72). We found that a $\Delta N_{\text{e}}^{\text{crit}}$ of 16.0 nmol ${\text{g}}_{\text{cdw}}^{-1}$ h^−1^ leads to a minimal sum of errors between predicted and observed growth, substrate uptake, and acetate secretion rates. The corresponding comparisons of experimentally determined and predicted phenotypes as well as flux distributions are shown in Fig. 8 and 9, respectively.

### Implementation.

All conducted simulations, model reconstructions, and data analyses were performed in MATLAB 2018a on a Windows 7 machine with 16 GB of RAM and an AMD FX-8350 eight-core (at 4.00 GHz) processor. COBRA toolbox functions (84) and the Gurobi Optimizer (8.0.0; Gurobi Optimization, Inc.) were utilized to process and solve the metabolic models. All MATLAB functions necessary to handle and build a protein allocation model (PAM) from a COBRA format-based, stoichiometric reconstruction are provided on GitHub (https://github.com/Spherotob/PAM_public).

## Supplementary Material

Reviewer comments
