# Supplementary material for: Proteome Regulation Patterns Determine Escherichia coli Wild-Type and Mutant Phenotypes
Source: mSystems. 2021 Mar 9;6(2):e00625-20. doi: 10.1128/mSystems.00625-20 (PMC8546978; doi:10.1128/mSystems.00625-20)
Supplement: TABLE S1 [file msystems.00625-20-st001.docx]

| **Substrate** | **Maximum uptake rate [mmol/gCDW/h]** |
| --- | --- |
| Glucose (aerobic) | 9.8 |
| Glucose (aerobic) | 20.3 |
| Galactose | 9.4 |
| Glycerol | 14.9 |
| Pyruvate | 53 |
| Xylose (aerobic) | 14.2 |
| Xylose (micro-aerobic) | 26.6 |
| Fructose | 9.6 |
| Fumarate | 26.5 |
| Acetate | 19.6 |
| Gluconate | 9.5 |
| Succinate | 24 |
| Mannose | 6.4 |
| Glucosamine | 9.5 |
